# Effect sizes for nonparametric tests

**DOI:** 10.11613/BM.2026.010101

**Published:** 2025-12-15

**Authors:** Fernanda Fiel Peres

**Affiliations:** Independent researcher, São Paulo, Brazil

**Keywords:** biostatistics, effect size, nonparametric statistics, writing in science

## Abstract

Effect size measures are important complements to P values, providing information about the magnitude and practical relevance of research findings. While widely discussed in the context of parametric tests, effect size estimation for nonparametric tests remains less explored. This article reviews standardized effect size measures applicable to four common nonparametric tests: Mann-Whitney, Wilcoxon signed-rank, Kruskal-Wallis, and Friedman. Commonly suggested classifications for these effect sizes are also discussed. This article aims to support researchers in reporting and interpreting effect sizes more effectively in nonparametric contexts.

## Graphical abstract

**Figure:**
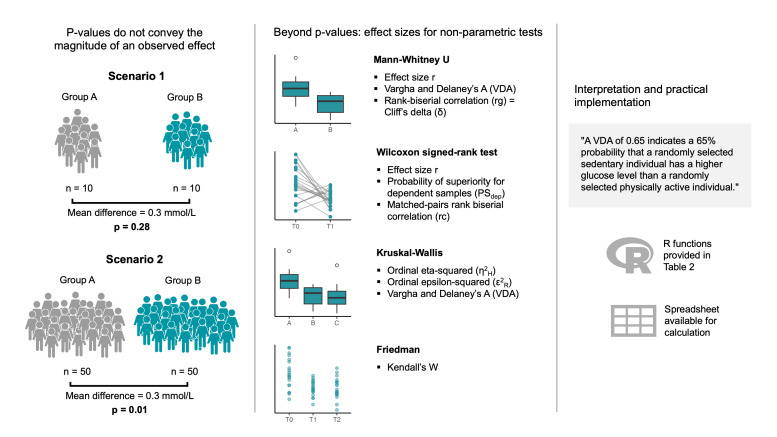

## Highlights

• Reporting effect sizes is essential to complement P values

• The article reviews effect size measures for four nonparametric tests

• Guidance is given on how to classify the magnitude of effect sizes

• Practical examples illustrate how to calculate and interpret effect sizes

## Why report effect sizes?

Scientific findings are often evaluated solely based on P values, despite the well-known limitations of this metric (*1*, *2*). A major concern is that P values do not convey the magnitude of an observed effect and are highly sensitive to sample size (*3*, *4*). For example, when comparing fasting glucose between sedentary and active individuals, a mean difference of 0.3 mmol/L is not statistically significant with 10 participants *per* group (P value = 0.278), but becomes significant when the group size is increased to 50 (P value = 0.014) - even though the means and standard deviation (0.6 mmol/L) remain identical (Table 1). This demonstrates that statistically significant results may correspond to trivial effects, whereas non-significant findings may hide potentially relevant differences. To address these limitations, it is widely recommended that effect sizes be reported alongside P values (*1*, *2*, *5*).

**Table 1:** Example of P value dependence on sample size

Scenario	N *per* group	Mean fasting glucose (mmol/L), sedentary group	Mean fasting glucose (mmol/L), exercise group	Pooled standard deviation (mmol/L)	Mean difference (mmol/L)	P
1	10	5.8	5.5	0.6	0.3	0.278
2	50	5.8	5.5	0.6	0.3	0.014

Effect sizes quantify the magnitude of an effect - in the example above, the difference between group means (0.3 mmol/L) is an unstandardized effect size, expressed in physical units (mmol/L). Such measures allow researchers to judge whether an observed difference is of practical or clinical importance. Standardized effect sizes, in contrast, have no units and facilitate comparisons across studies that use different measurement scales (*1*). A common standardized effect size for comparing two independent groups is Cohen’s d, calculated as the difference between group means divided by the pooled standard deviation (*6*). In the fasting glucose example (Table 1), Cohen’s d would be 0.50, conventionally classified as a medium effect (*7*). For nonparametric analyses, standardized effect sizes such as the probability of superiority, ordinal eta-squared, or Kendall’s W can be used to convey the magnitude of the effect without relying on units. These measures provide a way to quantify effect size when assumptions of parametric tests are not met.

Although reporting standardized effect sizes is relatively common in clinical research, it remains less frequent in basic science studies, including cell culture and animal experiments (*8*, *9*). In these fields, statistical analyses still rely heavily on P values, and the omission of effect size measures makes it difficult to determine whether non-significant results truly indicate absence of an effect or are instead a consequence of insufficient statistical power.

While standardized effect size measures for parametric tests are well documented in the literature, their application in nonparametric analyses has received comparatively limited attention (*6*, *10*, *11*). This paper addresses this gap by presenting effect size measures for four widely used nonparametric tests - Mann-Whitney U, Wilcoxon signed-rank, Kruskal-Wallis, and Friedman - and by reviewing suggested classification thresholds. It should be emphasized that such classifications are only heuristics; statistical descriptors such as “small,” “medium,” or “large” do not necessarily align with clinical or practical relevance (*12*, *13*). For instance, conventional guidelines consider a Cohen’s d of 0.8 a large effect (*7*). Yet this value may correspond to a mere 0.2 mmol/L difference in fasting glucose between groups when the pooled standard deviation is 0.25 mmol/L - a difference that might appear statistically impressive but be clinically negligible in many contexts.

## Mann-Whitney U test

The Mann-Whitney U test - also known as the Wilcoxon-Mann-Whitney test or Wilcoxon rank-sum test - compares the distributions of two independent groups. It is used when the independent variable (*i.e.*, the grouping variable) has two categories. The dependent variable may be either numerical or ordinal in nature (*14*).

### Effect size r

The most reported effect size after conducting a Mann-Whitney U test is the effect size r. Its calculation is straightforward and requires only the values of z and N. Here, z is the standardized test statistic from the Mann-Whitney U test, which is provided by most statistical software packages. If unavailable, the z value can be calculated manually using the equation (Eq.) provided below (*15*).



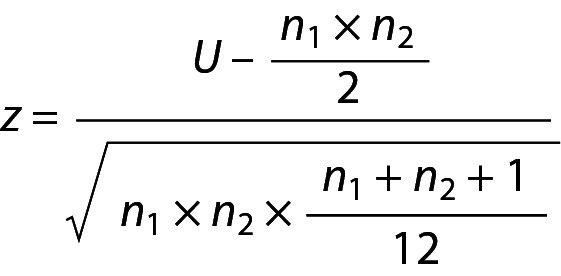



In this formula, U is the Mann-Whitney U statistic, and n_1_ and n_2_ are the sample sizes of the two independent groups. This version does not include continuity correction or tie adjustments. For details on applying these in the z calculation, see Zar (*16*).

Once the z value is obtained, the effect size r is calculated as (Eq. 2):



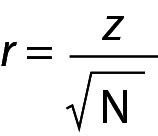



where N = n_1_ + n_2_ is the total sample size.

The absolute value of r is commonly classified as small (≥ 0.1), medium (≥ 0.3), or large (≥ 0.5), following Cohen’s thresholds (*7*, *17*). Other classifications with finer gradations have also been proposed (*18*, *19*).

For example, consider two groups with n_1_ = n_2_ = 10 and a Mann-Whitney test yielding U = 35. From the formula above, this corresponds to z = - 1.13 and hence r = - 0.25. According to Cohen’s thresholds (*7*), this represents a small effect size.

Table 2 lists the R functions used to compute r and other effect sizes (*20*). In addition, an Excel spreadsheet is provided as a supplementary tool to facilitate their calculation.

**Table 2:** Summary of effect size measures for nonparametric tests with corresponding R functions

**Test**	Corresponding parametric test*	**Effect size**	**Formula/ Method**	**R function / Package**	**Suggested classification^†^**
Mann-Whitney U test	Independent t-test	r	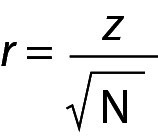	wilcoxonR() from rcompanion	Small: |r| ≥ 0.1 Medium: |r| ≥ 0.3 Large: |r| ≥ 0.5 Ranges from -1 to 1, with 0 indicating no difference between groups.
Mann-Whitney U test	Independent t-test	Vargha and Delaney’s A (VDA)	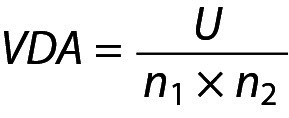	vda() from rcompanion	Small: VDA ≥ 0.56 Medium: VDA ≥ 0.64 Large: VDA ≥ 0.71 Ranges from 0 to 1, with 0.5 indicating no difference between groups.
Mann-Whitney U test	Independent t-test	Rank-biserial correlation coefficient (rg) = Cliff’s delta (δ)	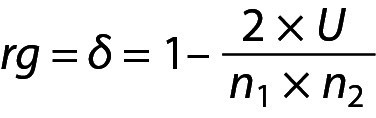	wilcoxonRG() from rcompanion rank_biserial() from effectsize	Small: |rg| ≥ 0.11 Medium: |rg| ≥ 0.28 Large: |rg| ≥ 0.43 Ranges from -1 to 1, with 0 indicating no difference between groups.
Wilcoxon signed-rank test	Paired t-test	r	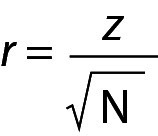	wilcoxonPairedR() from rcompanion	Small: |r| ≥ 0.1 Medium: |r| ≥ 0.3 Large: |r| ≥ 0.5 Ranges from -1 to 1, with 0 indicating no difference between groups.
**Test**	Corresponding parametric test*	**Effect size**	**Formula/ Method**	**R function / Package**	**Suggested classification^†^**
Wilcoxon signed-rank test	Paired t-test	Matched-pairs rank biserial correlation coefficient (rc)	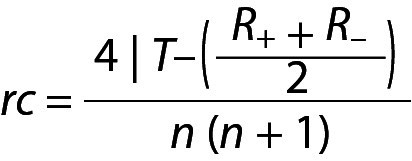	wilcoxonPairedRC() from rcompanion rank_biserial(paired = TRUE) from effectsize	Small: |rc| ≥ 0.11 Medium: |rc| ≥ 0.28 Large: |rc| ≥ 0.43 Ranges from -1 to 1, with 0 indicating no difference between groups.
Wilcoxon signed-rank test	Paired t-test	Probability of superiority for dependent groups (PS_dep_)	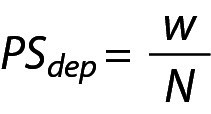 Versions that account for ties are discussed in the main text.	/	Small: PS_dep_ ≥ 0.56 Medium: PS_dep_ ≥ 0.64 Large: PS_dep_ ≥ 0.71 Ranges from 0 to 1, with 0.5 indicating no difference between groups.
Kruskal-Wallis test	One-way ANOVA	Ordinal eta-squared (η^2^_H_)	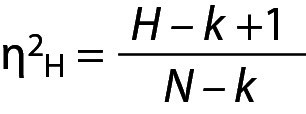	ordinalEtaSquared() from rcompanion rank_eta_squared() from effectsize	Small: η^2^_H_ ≥ 0.01 Medium: η^2^_H_ ≥ 0.06 Large: η^2^_H_ ≥ 0.14 Ranges from 0 to 1, with higher values indicating a larger effect.
Kruskal-Wallis test	One-way ANOVA	Ordinal epsilon-squared (ε^2^_R_)	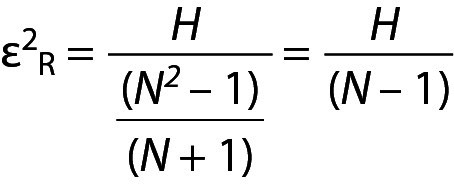	epsilonSquared() from rcompanion rank_epsilon_squared() from effectsize	Small: ε^2^_R_ ≥ 0.01 Medium: ε^2^_R_ ≥ 0.06 Large: ε^2^_R_ ≥ 0.14 Ranges from 0 to 1, with higher values indicating a larger effect.
Kruskal-Wallis test	One-way ANOVA	Vargha and Delaney’s A (VDA)	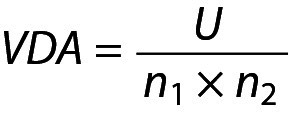 Should be calculated from the Mann-Whitney U test for each pairwise comparison.	multiVDA() from rcompanion	Small: VDA ≥ 0.56 Medium: VDA ≥ 0.64 Large: VDA ≥ 0.71 Ranges from 0 to 1, with 0.5 indicating no difference between groups.
Friedman test	Repeated- measures ANOVA	Kendall’s W	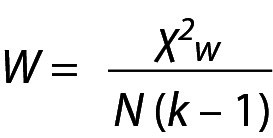	kendallW() from rcompanion kendalls_w() from effectsize friedman_effsize() from rstatix	Small: W ≥ 0.1 Medium: W ≥ 0.3 Large: W ≥ 0.5 Ranges from 0 to 1, with higher values indicating a larger effect.
*The parametric tests shown are not exact counterparts of the nonparametric tests, as their null hypotheses differ: the parametric tests assess mean differences, while the nonparametric tests assess differences in overall distributions. ^†^References for each of these classifications, as well as alternative classification criteria, are provided in the main text.					

### Vargha and Delaney’s A effect size or probability of superiority

After conducting a Mann-Whitney U test, researchers can compute Vargha and Delaney’s A (VDA) statistics, a variant of the Common Language Effect Size (CLES), also known as the probability of superiority (*21*, *22*). This metric offers an intuitive interpretation: it represents the probability that a randomly selected subject from one group will have a higher observed value than a randomly selected subject from the other group.

Vargha and Delaney’s A is calculated using the formula below (Eq. 3), where U is the Mann-Whitney U statistic, and n_1_ and n_2_ are the sample sizes of groups 1 and 2, respectively (*22*):



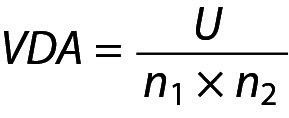



Vargha and Delaney’s A ranges from 0 to 1, with 0.5 indicating no difference between groups. Values closer to 1 (or 0) indicate stronger superiority of one group over the other. According to Vargha and Delaney, VDA values can be classified as small (VDA ≥ 0.56), medium (VDA ≥ 0.64), or large (VDA ≥ 0.71) (*21*). The corresponding R function for computing VDA is shown in Table 2.

For the fasting glucose example presented earlier, consider two groups of 10 participants each, with a Mann-Whitney test yielding U = 35. In this case, the corresponding VDA is 0.35. Because this value is below 0.5, we interpret it as 1 − 0.35 = 0.65. This indicates a 65% probability that a randomly selected sedentary individual has a higher glucose concentration than a randomly selected physically active individual. This interpretation offers a more intuitive understanding of the group difference, complementing the information provided by P values.

### Rank-biserial correlation coefficient or Cliff’s delta

The rank-biserial correlation coefficient (also referred to as Glass’ rank-biserial correlation) is commonly abbreviated as r or rg (*23*). In the context of the Mann–Whitney U test, rg is equivalent to Cliff’s delta (δ) effect size (*24*). These effect sizes are linear transformations of VDA statistics and extend the range of possible values from −1 to 1.

A value of 0 for either rg or δ indicates no difference between the two groups. A value of 1 signifies that all observations in Group 1 exceed those in Group 2, whereas a value of −1 indicates that all observations in Group 2 exceed those in Group 1 (*23*). According to Vargha and Delaney, the absolute value of δ can be interpreted as small (≥ 0.11), medium (≥ 0.28), or large (≥ 0.43) (*21*). However, this classification is not universally accepted. Tomczak and Tomczak, for instance, recommend applying the thresholds proposed by Cohen for Pearson’s r: small (≥ 0.1), medium (≥ 0.3), and large (≥ 0.5) (*7*, *25*).

The formula used to calculate rg (or δ) is presented below (Eq. 4 and Eq. 5), where U denotes the Mann-Whitney U statistic, and n_1_ and n_2_ represent the sample sizes of groups 1 and 2, respectively (*21*). The R function used to compute rg is provided in Table 2.



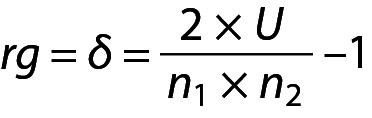



*rg = δ =* 2 × *VDA* – 1

For the same fasting glucose example, with a VDA of 0.65, the corresponding rank-biserial correlation (rg) is 0.30, indicating a moderate (medium) effect and a tendency for higher glucose concentrations in the sedentary group compared with the physically active group.

## Wilcoxon signed-rank test

Wilcoxon signed-rank test compares the scores of two dependent (paired) groups. It is appropriate when the independent variable has two categories referring to related groups, such as before and after an intervention.

The procedure begins by calculating, for each subject, the difference between the two paired measurements. These differences are then ranked according to their absolute values, and the test statistic is obtained by comparing the sum of the positive ranks with the sum of the negative ranks (for a detailed explanation of the manual computation, see King *et al.*) (*14*).

The Wilcoxon signed-rank test is often described as suitable for either numeric or ordinal dependent variables. However, King *et al.* caution that applying it to ordinal data requires assumptions that are frequently unrealistic (*14*). Specifically, it assumes that the differences between pairs of scores can be meaningfully ranked and that the scale intervals are consistent. For example, in a pain scale from 1 to 10 (ordinal), the test assumes that the difference between pain levels 4 and 5 is equivalent to the difference between levels 8 and 9 - an assumption that may not always hold (*14*).

### Effect size r

The calculation of the r effect size for the Wilcoxon signed-rank test follows the same formula as for the Mann-Whitney test (*17*). In this context, z is the standardized test statistic from the Wilcoxon signed-rank test, usually reported by statistical software but also obtainable through manual calculation (*14*, *15*). The standardized z can be computed as:



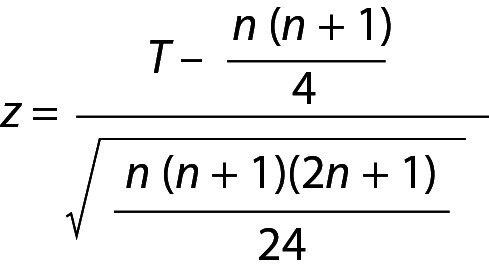



where T is the smaller of the sums of positive ranks (R_+_) and negative ranks (R_-_), and n is the number of non-zero differences. This formula for z does not include continuity correction or tie adjustments (see Zar for these versions) (*16*). Once the z value is obtained, the effect size r is calculated using the formula below (Eq. 7) (*17*).



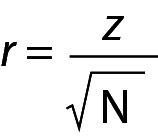



According to Cohen’s guidelines, the absolute values of r can be interpreted as small (≥ 0.10), medium (≥ 0.30), or large (≥ 0.50), as also noted by Fritz *et al.* (*7*, *17*). The R function used to compute r is provided in Table 2.

### Probability of superiority for dependent groups

The probability of superiority for dependent groups (PS_dep_) follows the same interpretative framework as the probability of superiority for independent groups (VDA). Probability of superiority for dependent groups represents the probability that a randomly selected experimental subject exhibits a positive (or negative, depending on the direction of interest) difference between paired observations. Its values range from 0 to 1, where 0.5 indicates no difference between groups (*22*).

The calculation of PS_dep_ depends on the presence of ties - cases in which experimental subjects have identical values in both conditions, resulting in a difference of zero. When no ties are present, PS_dep_ can be computed using the formula below (Eq. 8) where w denotes the count of differences in the direction of interest (positive or negative), and N is the sample size (number of paired observations) (*22*).



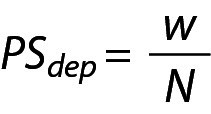



In the presence of ties, the calculation of PS_dep_ must be adjusted. According to Grissom and Kim, two approaches are possible: (*1*) exclude ties from the calculation; or (*2*) include half the number of ties in the numerator (*22*). The authors recommend reporting both calculations in articles (*22*).

First option (excluding ties):



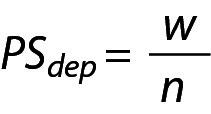



where n is number of pairs without ties.

Second option (including ties):



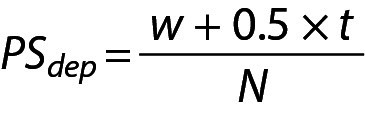



where t is the number of pairs with ties, and N is the total number of pairs.

The VDA, explained earlier in relation to independent groups, corresponds to the probability of superiority with adjustment for ties (*21*). Therefore, the classification thresholds for PS_dep_, whether ties are absent or corrected for, can follow those suggested for VDA: small effect (PS_dep_ ≥ 0.56), medium effect (PS_dep_ ≥ 0.64), and large effect (PS_dep_ ≥ 0.71) (*21*).

It is important to note that the term probability of superiority for dependent samples and its notation PS_dep_ are not universally standardized; different authors may refer to the same effect size using different terms.

### Matched-pairs rank biserial correlation coefficient

The matched-pairs rank biserial correlation coefficient is abbreviated as r or rc (*14*, *23*). As with the version for independent samples (rg), rc ranges from -1 to 1. A value of 1 indicates that all differences between groups 1 and 2 are positive (group 2 > group 1), whereas a value of −1 indicates that all differences are negative (group 1 > group 2) (*23*). The paired rank-biserial correlation coefficient can be calculated using the formula below (Eq. 11) (*14*):



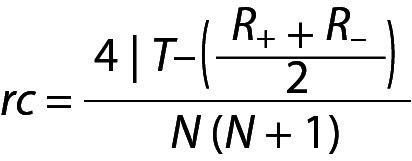



where R_+_ represents the sum of positive ranks, R_-_ the sum of negative ranks, T the smallest value between R+ and R-, and N = sample size (number of pairs).

Tomczak and Tomczak recommend interpreting the absolute values of rc using the same thresholds as Pearson’s correlation coefficient, as discussed above (*25*). Alternatively, it can be classified according to Vargha and Delaney’s guidelines for the independent-samples rank-biserial correlation: small (rc ≥ 0.11), medium (rc ≥ 0.28), or large (rc ≥ 0.43) (*21*). The R function used to compute rc is provided in Table 2.

As an illustration, based on the fasting glucose data (mmol/L) in Table 3, the Wilcoxon signed-rank test yielded T = 21.0 with N = 10 pairs. The standardized statistic was z = - 0.66, which corresponds to the following effect sizes: r = - 0.209 and rc = 0.236, both interpreted as small effects. The PS_dep_, computed using the number of negative differences, was 60%. This indicates a 60% probability that a randomly selected individual from this sample shows a decrease from before to after.

**Table 3:** Intermediate Calculations for Effect Size Estimation in the Wilcoxon Signed-Rank Test (Fasting Glucose, mg/dL).

**ID**	**Before (mmol/L)**	**After (mmol/L)**	**Difference (After - Before)**	**|Difference|**	Rank of |Difference|*
A	5.8	5.6	-0.2	0.2	5.5
B	5.5	5.6	0.1	0.1	2
C	7.2	6.8	-0.4	0.4	9
D	6.2	6.1	-0.1	0.1	2
E	6.9	7.1	0.2	0.2	5.5
F	5.8	5.6	-0.2	0.2	5.5
G	6.2	5.4	-0.8	0.8	10
H	7.4	7.7	0.3	0.3	8
I	5.7	5.6	-0.1	0.1	2
J	5.4	5.6	0.2	0.2	5.5
Sum of positive ranks = 21 Sum of negative ranks = 34 T = 21 Number of positive differences = 4 Number of negative differences = 6 Number of ties = 0					
* In case of ties, average ranks are assigned. For example, the smallest absolute difference is 2, which occurs four times; thus, each observation receives the average of ranks 1, 2, and 3, resulting in a rank of 2.					

## Effect sizes for Kruskal-Wallis test

The Kruskal-Wallis test is an extension of the Mann-Whitney U test for situations involving more than two groups. The Kruskal-Wallis test is appropriate when the independent variable is nominal with more than two categories, and the dependent variable is either numerical or ordinal (*14*).

### Ordinal eta-squared (η^2^_H_)

An effect size commonly used for the Kruskal-Wallis test is the eta-squared calculated from the test statistic H, often referred to as ordinal eta-squared (η^2^_H_). This effect size is computed using the following formula (*25*, *26*):



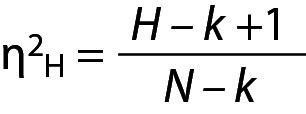



where H represents the Kruskal-Wallis test statistic, k is the number of groups and N the total number of observations across all groups.

Ordinal eta-squared should not be confused with the eta-squared effect size (η^2^) derived from ANOVA (*6*). The ANOVA eta-squared is calculated from the F-statistic and degrees of freedom and represents the proportion of variance in the dependent variable explained by the independent variable (*6*). In contrast, ordinal eta-squared is based on the H-statistic from the Kruskal-Wallis test. To distinguish it from the ANOVA version, it is termed ordinal eta-squared and denoted as η^2^_H_, following Cohen’s notation (*26*).

Values of ordinal eta-squared range between 0 and 1. According to Cohen and Tomczak and Tomczak, when multiplied by 100, η^2^_H_ can be interpreted as the percentage of variance in the dependent variable explained by the independent variable (*25*, *26*). However, this interpretation has been criticized because the concept of variance does not strictly apply to rank-based analyses such as the Kruskal-Wallis test (*27*). To illustrate, consider three independent samples: Sample 1 = 10, 11, 12; Sample 2 = 10, 50, 51; Sample 3 = 10, 200, 201. While the original variances differ greatly (1, 547, and 12097, respectively), ranking the values within each sample results in identical ranks (*1*-*3*) with the same mean and variance for all three samples. This demonstrates that rank-based variance captures relative order rather than absolute differences. Consequently, interpreting η^2^_H_ percentage of variance explained may be misleading, as it ignores the magnitude of differences in the original measurements.

The conventional classification of η^2^_H_ effect sizes follows the same thresholds used for ANOVA eta-squared (η^2^): small effect (≥ 0.01), medium effect (≥ 0.06), and large effect (≥ 0.14) (*7*, *28*). Functions in R for calculating ordinal eta-squared are listed in Table 2.

### Ordinal epsilon-squared (ε^2^_R_)

Epsilon-squared calculated from the Kruskal-Wallis test statistic H - referred to as ordinal epsilon-squared (ε^2^_R_) - is another suggested effect size for this test. This measure can be calculated using the following formula (Eq. 13), simplified by the author (*14*, *25*):



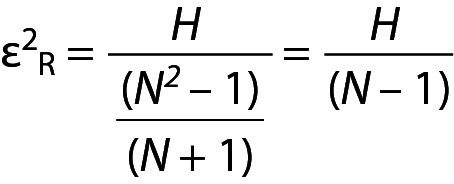



Similar to ordinal eta-squared, ordinal epsilon-squared ranges from 0 to 1. Values near zero indicate no association between the dependent and independent variables, while values equal to 1 represent a perfect association (*14*).

It is important to avoid confusing ordinal epsilon-squared-denoted as ε^2^_R_, following King *et al.* - with the non-ordinal epsilon-squared (ε^2^) calculated from ANOVA F-values and degrees of freedom (*6*, *14*). The ANOVA epsilon-squared (ε^2^) is a bias-corrected version of eta-squared (η^2^) (*29*).

There is no consensus regarding the classification thresholds for ordinal epsilon-squared. Since epsilon-squared is an adjusted version of eta-squared, some authors recommend using the same classification criteria as for eta-squared (*30*). Alternatively, based on its equivalence to the effect size r, another suggested classification is small effect (ε^2^_R_ ≥ 0.01), medium effect (ε^2^_R_ ≥ 0.08), or large effect (ε^2^_R_ ≥ 0.26) (*31*). Functions in R for calculating ordinal epsilon-squared are also presented in Table 2.

To illustrate, consider fasting glucose concentrations in three groups (N = 10 each): sedentary, moderately active, and highly active. The Kruskal–Wallis test yields H = 8.4. Applying the above formulas gives η^2^_H_ = 0.237 and ε^2^_R_ = 0.290, both interpreted as large effects.

### Ordinal epsilon-squared versus ordinal eta-squared

As explained in the previous section, the classical epsilon-squared effect size (ε^2^) from ANOVA is a bias-corrected version of the eta-squared effect size (η^2^) (*29*). In parametric one-way ANOVA models, η^2^ corresponds to the R^2^ value of the model, while ε^2^ corresponds to the adjusted R^2^ (*30*). However, this direct relationship does not apply to their ordinal counterparts, η^2^_H_ and ε^2^_R_, which are calculated from ranked data.

There is currently no empirical research directly comparing η^2^_H_ and ε^2^_R_. Notably, Tao raised the possibility - in a discussion forum - that the formulas for these two effect sizes may have been inadvertently switched in the literature (*32*). This hypothesis arises from the observation that, when conducting an ANOVA on ranked data, the unadjusted R^2^ aligns with ε^2^_R_, whereas the adjusted R^2^ corresponds to η^2^_H_.

This correspondence can be demonstrated mathematically. In a one-way ANOVA with k groups and N observations, the linear model on ranked data includes an intercept and p = k−1 predictors. Consider the formula for the adjusted R^2^ (R^2^_adj_):



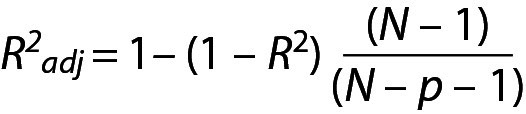



Substituting p = k−1, we have:



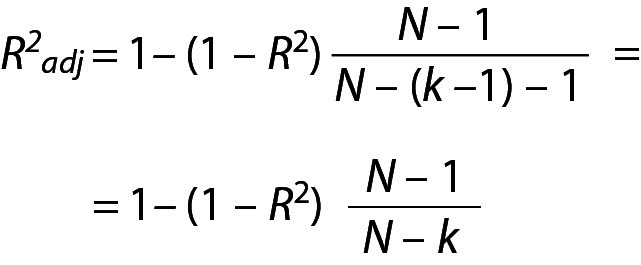



Replacing R^2^ with the ε^2^_R_ formula presented in the previous section, we obtain:



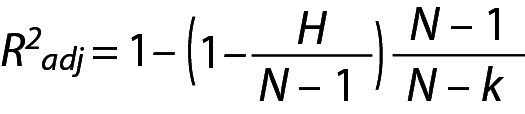





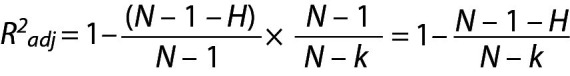





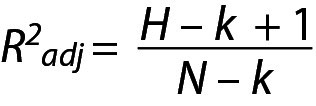



Thus, under these assumptions, if ε^2^_R_ = R^2^, then η^2^_H_ = R^2^_adj_. The mathematical equivalence between R^2^ in a regression on ranked data and ε^2^_R_ is shown in (*33*).

It is important to stress that this inversion is speculative and has not been formally validated. Therefore, researchers should continue to apply the formulas established in the current literature and outlined in previous sections. To reduce ambiguity, it is recommended that authors explicitly report the exact formula used for calculating the effect size.

Moreover, because η^2^_H_ and ε^2^_R_ are calculated from ranked data, their interpretation as proportions of explained variance is conceptually debatable and has been subject to criticism (*27*). This limitation complicates their practical use. As a result, alternative effect size measures - such as VDA - may provide a more robust interpretation for ordinal data and will be discussed in the subsequent section.

### VDA effect size (probability of superiority)

As discussed earlier in relation to the VDA effect size for the Mann-Whitney U test, the VDA corresponds to the probability of superiority when comparing two groups (*21*, *22*). When performing a Kruskal-Wallis test, one option is to calculate the VDA effect size for all possible pairwise comparisons between groups (*31*). To manually calculate these VDA effect sizes, the researcher must conduct Mann-Whitney U tests for each pair of groups and then compute the VDA value using the formula previously described for the Mann-Whitney U test. The “multiVDA” function from the “rcompanion” package enables the calculation of VDA effect sizes for all pairwise comparisons simultaneously, streamlining the analysis process (*34*). This function is included in Table 2.

## Friedman test

The Friedman test is a non-parametric method used to detect differences in treatments across multiple related groups or repeated measurements on the same subjects. It is commonly regarded as the nonparametric alternative to the repeated measures ANOVA (*14*). This test is appropriate when the independent variable involves related groups with more than two categories, and the dependent variable is either numeric or ordinal (*14*, *15*).

### Kendall’s W effect size

For the Friedman test, Kendall’s W coefficient (also known as the coefficient of concordance) is the primary effect size measure recommended (*14*, *25*). Kendall’s W can be calculated using the following formula (*14*, *25*):



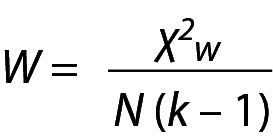



where χ_w_^2^ is the chi-square statistic from the Friedman test, k the number of related groups or conditions and N the total sample size.

The value of W ranges from 0 to 1, with higher values indicating a stronger effect. There is no universal consensus on the classification of Kendall’s W effect size. Some authors recommend applying Cohen’s guidelines for w, with thresholds for small (W ≥ 0.1), medium (W ≥ 0.3), or large (W ≥ 0.5) effects (*7*, *35*). Alternatively, Mangiafico proposes classifications that depend on the number of related groups (k) (*31*). Ben-Shachar suggests adopting the same classification system used for the kappa concordance coefficient, with the most widely used scale being that of Landis and Koch, which defines slight agreement (W ≥ 0.0), fair (W ≥ 0.2), moderate (W ≥ 0.4), substantial (W ≥ 0.6), and almost perfect agreement (W ≥ 0.8) (*30*, *36*).

To illustrate, consider fasting glucose measured across three conditions in the same 10 participants: morning, afternoon, and evening. The Friedman test yields χ_w_^2^ = 6.5. Given the study design with N = 10 and k = 3, the corresponding W = 0.325, which is classified as a medium effect.

## Effect sizes *versus* sample size

The sample size required in a study depends, among other factors, on the effect size that one aims to detect. Smaller effects generally require larger samples to achieve adequate statistical power, whereas larger effects can be detected with fewer observations. For instance, to detect a mean difference of 0.3 mmol/L in fasting glucose between two groups (assuming a standard deviation of 0.6 mmol/L, 80% power, and a 5% significance level), 64 participants *per* group would be needed. In contrast, detecting a 0.6 mmol/L difference under the same conditions requires 17 participants *per* group (*7*).

Calculating the required sample size for nonparametric tests is generally less straightforward than for parametric tests, as it depends on the characteristics of the distribution of the underlying data (*37*). Some guidelines suggest using the sample size calculated for the corresponding parametric test and increasing it by 15% - but this rule-of-thumb is based on practical considerations rather than a formal consensus (*38*). A more robust and reproducible alternative is to use software such as the open-source G*Power, which can perform power analyses tailored to specific nonparametric scenarios, considering standardized effect sizes, significance level, and desired power (see 37 for detailed guidance) (*39*).

Although effect sizes are theoretically independent of sample size, there is ongoing debate about this assumption. Recent evidence indicates that observed effect sizes tend to be larger in studies with smaller samples, potentially reflecting publication bias favoring significant results (*40*, *41*). This highlights the importance of reporting effect sizes alongside p-values and considering both the effect magnitude and the sample size when interpreting study findings. Researchers should exercise caution when comparing effect sizes across studies with very different sample sizes, as apparent differences may reflect methodological or reporting biases rather than true differences in effects.

## Data Availability

No data was generated during this study, so data sharing is not applicable to this article.
